# Relationship of Hyperglycaemia, Hypoglycaemia, and Glucose Variability to Atherosclerotic Disease in Type 2 Diabetes

**DOI:** 10.1155/2018/7464320

**Published:** 2018-07-22

**Authors:** Caroline Jane Magri, Dillon Mintoff, Liberato Camilleri, Robert G. Xuereb, Joseph Galea, Stephen Fava

**Affiliations:** ^1^Department of Cardiology, Mater Dei Hospital and University of Malta, Msida, Malta; ^2^Department of Cardiology, Mater Dei Hospital, Msida, Malta; ^3^Mater Dei Hospital, Msida, Malta; ^4^Statistics & Operations Research, Faculty of Science, University of Malta, Msida, Malta; ^5^Mater Dei Hospital and University of Malta, Msida, Malta

## Abstract

**Objective:**

Type 2 diabetes mellitus (T2DM) is known to be associated with increased cardiovascular risk. The aim of this study was therefore to investigate the independent effects of hyperglycaemia, hypoglycaemia, and glucose variability on microvascular and macrovascular disease in T2DM.

**Methods:**

Subjects with T2DM of <10 years duration and on stable antiglycaemic treatment underwent carotid intima-media thickness (CIMT), ankle-brachial index (ABI), albumin-creatinine ratio (ACR), and HbA_1c_ measurement, as well as 72-hour continuous glucose monitoring. Macrovascular disease was defined as one or more of the following: history of ischaemic heart disease (IHD), cerebrovascular accident (CVA), ABI < 0.9, or abnormal CIMT.

**Results:**

The study population comprised 121 subjects with T2DM (89 males : 32 females). The mean age was 62.6 years, and the mean DM duration was 3.7 years. Macrovascular disease was present in 71 patients (58.7%). In multivariate logistic regression analysis, body surface area (BSA) (OR 18.88 (95% CI 2.20–156.69), *p* = 0.006) and duration of blood glucose (BG) < 3.9 mmol/L (OR 1.12 (95% CI 1.014–1.228), *p* = 0.024) were independent predictors of macrovascular disease. BSA (OR 12.6 (95% CI 1.70–93.54), *p* = 0.013) and duration of BG < 3.9 mmol/L (OR 1.09 (95% CI 1.003–1.187), *p* = 0.041) were independent predictors of abnormal CIMT. Area under the curve for BG > 7.8 mmol/L (*β* = 15.83, *p* = 0.005) was the sole independent predictor of albuminuria in generalised linear regression.

**Conclusions:**

This study demonstrates that hypoglycaemia is associated with the occurrence of atherosclerotic disease while hyperglycaemia is associated with microvascular disease in a Caucasian population with T2DM of recent duration.

## 1. Introduction

Diabetes is known to be associated with both microvascular and macrovascular disease [1]. Whilst good glycaemic control has been consistently shown to reduce microvascular disease, most intervention studies have failed to document a reduction in macrovascular disease outcomes [2, 3].

One possible explanation for this discrepancy is the detrimental effect of hypoglycaemia. Östgren and colleagues have recently reported a U-shaped relationship of HbA_1c_ with cardiovascular events, including mortality [4]. A recent meta-analysis has also confirmed that hypoglycaemia is associated with a twofold increased risk of cardiovascular disease in type 2 diabetes [5]. Furthermore, both hyperglycaemia and hypoglycaemia following acute coronary syndrome have been associated with increased mortality [6, 7] The traditional explanations of these observations are the proarrhythmogenic effect of hypoglycaemia as a consequence of catecholamine release and QTc prolongation [8, 9] and the unavailability of an energy substrate to the myocardium during hypoglycaemia leading to an ischaemia equivalent [10]. However, the increased mortality extends beyond the hypoglycaemic episode itself. Although a hypoglycaemic episode is known to predict future ones [11], this is unlikely to be the sole mechanism. Another possible contributory mechanism that has not been well investigated is the possibility that hypoglycaemia may accelerate atherosclerosis, as suggested by some animal studies. For example, Yasunari and colleagues reported that repeated hypoglycaemia worsens injury-provoked intimal thickening in a male Goto-Kakizaki rat carotid artery [12], whilst Jin and colleagues reported that hypoglycaemia induced monocyte adhesion to rat aortic endothelium [13]. Furthermore, a small study found higher carotid and femoral intima-media thickness in 25 subjects with type 1 diabetes and with repeated hypoglycaemic episodes when compared to 20 subjects with type 1 diabetes but without hypoglycaemia [14]. However, continuous glucose monitoring was not performed, and therefore, hyperglycaemic episodes were not captured. Since the two groups had similar HbA_1c_, it is likely that the hypoglycaemic group also had more hyperglycaemia, which might conceivably have contributed to increased intima-media thickness.

Another possible explanation of the failure of clinical trials to demonstrate a beneficial effect of glycaemic control is that none of them targeted reducing fluctuations in blood glucose. Indeed, many forms of treatment may increase blood glucose fluctuations. Acute fluctuations in blood glucose have been shown to be associated with increased mortality [15]. This may be mediated by endothelial dysfunction, oxidative stress, and endothelial cell apoptosis [16]. Such effects of glucose fluctuations could predispose to both microvascular and macrovascular disease.

The aim of this study was therefore to investigate the independent effects of hyperglycaemia, hypoglycaemia, and glucose variability on microvascular and macrovascular disease in type 2 diabetes. Urinary albumin-creatinine served as a marker of microvascular disease. Ankle-brachial index, carotid intima-media thickness, and documented cardiovascular disease served as markers of macrovascular disease. 72-hour continuous glucose monitoring was used to assess hypoglycaemia, hyperglycaemia, and glucose variability. HBA_1c_ and fructosamine served as additional glycaemic indexes.

## 2. Methods

The study was cross-sectional in nature; data collection was performed over a one-year period, from April 2015 to April 2016. Patients were randomly selected from the list of outpatients attending the Diabetes Centre, Mater Dei Hospital, Malta, during the year 2014. The inclusion criteria were subjects who were diagnosed with type 2 DM according to World Health Organization criteria within the last ten years and were on stable antiglycaemic treatment. Patients suffering from dementia or mental illnesses with the consequent inability to give an informed consent with regard to participation in the study were excluded from the study. Other exclusion criteria included recent admission to hospital with a diabetes-related complication such as hyperglycaemic hyperosmolar ketoacidosis, liver failure, or kidney failure, as well as any other factors that result in glycaemic fluctuations. The study protocol was approved by the University of Malta Research Ethics Committee. All participants gave written informed consent.

### 2.1. Clinical and Laboratory Measurements

The presence of history of ischaemic heart disease and of cerebrovascular disease was ascertained by use of a questionnaire and by review of medical notes. Height and weight were measured using a calibrated balance and a stadiometer, with the subjects wearing light clothing and without shoes. Body mass index (BMI) was calculated as weight divided by height squared. Waist circumference was measured to the nearest 0.5 cm in the horizontal plane at the midpoint between the lowest rib and the iliac crest [17]. The waist index was calculated as waist circumference (cm) divided by 94 for men and 80 for women [18] Office blood pressure was measured in the supine position after 5 min of rest.

All subjects underwent routine blood investigations in the fasting state on the day of the clinical examination. No medication was taken on the morning of the examination. Patients who were treated with insulin discontinued injections after 22:00 on the day preceding the examination. Estimated glomerular filtration rate (eGFR) was calculated using the Modified Diet in Renal Disease (MDRD) formula (National Kidney Foundation Calculator for Healthcare Professionals). Haemoglobin A_1c_ (measured using high-performance liquid chromatography) and fructosamine were taken as markers of glycaemic control. Urine albumin was measured by an immunoturbidometric technique (Roche Diagnostics, Mannheim, Germany). Creatinine was measured by a kinetic colorimetric test using the Jaffe reaction (Roche Diagnostics). Albumin-creatinine ratio (ACR) was assessed in all patients as a marker of microvascular disease. Body surface area was provided by the Esaote machine used to perform CIMT by inputting weight and height.

### 2.2. Continuous Glucose Monitoring

Seventy-two-hour subcutaneous continuous glucose monitoring (CGM) was performed in all study participants on an ambulatory basis using the iPro2 continuous glucose-monitoring system (Medtronic MiniMed, Northridge, CA, USA). Participants were advised to pursue their normal daily living without alterations in their usual diet, exercise, and medications taken. Data from the iPro2 were uploaded online via the CareLink iPro software. The following parameters were extracted for each patient: mean blood glucose (MBG), standard deviation of blood glucose (SD), the highest and lowest blood glucose values, the number of high and low excursions, the area under the curve when the blood glucose was higher than 7.8 mmol/L (AUC > 7.8), the area under the curve when the blood glucose was lower than 3.9 mmol/L (AUC < 3.9), the duration (%) when the blood glucose was higher than 7.8 mmol/L, the duration (%) when the blood glucose was lower than 3.9 mmol/L, and the duration (%) when the blood glucose was between 3.9 mmol/L and 7.8 mmol/L.

### 2.3. Carotid Intima-Media Thickness Measurement

Carotid intima-media thickness (CIMT) was assessed in both common carotid arteries in each participant using the Esaote Quality intima media thickness (QIMT®). The common carotid artery was utilised since, according to the Mannheim consensus, it increases the accuracy and reproducibility of the measurements obtained. The Esaote QIMT utilises a radio frequency signal to enable measurement of the CIMT with high spatial resolution. The QIMT tool automatically provides accurate measurements of all the parameters which are independent from both the investigator and device settings. Results were categorised as normal or abnormal using age-specific cut-offs, which have been validated in large worldwide databases [19]. Furthermore, all study participants were screened for the presence of carotid plaques in view that the combination of plaque area and thickness have greater value in predicting CVD compared to the thickness alone [17]. Patients with an increased CIMT as assessed by QIMT and/or presence of carotid plaque were categorised as having an abnormal CIMT.

### 2.4. Clinical Definitions

eGFR was defined as being low if it was <60 mL/min/1.73m^2^. PAD was defined as an ABI of ≤0.9. The mean arterial pressure was the average arterial pressure during a single cardiac cycle and pulse pressure the difference between the systolic and diastolic blood pressure readings. Macrovascular disease was defined as the presence of one or more of the following: history of ischaemic heart disease (IHD) or cerebrovascular accident (CVA), ABI < 0.9, or abnormal CIMT.

### 2.5. Statistical Analysis

All data were analysed using SPSS version 24.0 for Windows. Results are presented as mean ± standard deviation (SD) or median (interquartile range (IQR)). Continuous variables were checked for normality of distribution using the Kolmogorov-Smirnov method. Comparisons of continuous variables between two groups were made using independent sample *t*-test for normally distributed variables. For nonparametric variables, the Mann–Whitney *U* test was used for comparison of 2 groups. Categorical variables were compared using the *χ*^2^ test. The Pearson test was used to test correlation of normally distributed variables, and the Spearman test was used for comparison of non-normally distributed variables.

Univariate followed by multivariate analyses (logistic regression analyses) were performed to identify independent determinants of both the occurrence of macrovascular disease and abnormal CIMT in the study population. Variables were entered into the regression model if their *p* value was <0.1 in univariate analysis. Predictors were removed from the model if their *p* value exceeded 0.05.

With regard to ACR, univariate followed by multivariate analysis was again performed with variables with a *p* value < 0.1 in univariate analysis being included in the multivariate model; however, a generalised linear model was performed as multivariate analysis in view that ACR exhibited a gamma distribution (a right-skewed distribution). All tests were two-sided, and a *p* value of <0.05 was considered to be statistically significant.

Sample size was determined so as to have statistical power of 90% to detect a moderate effect size (*f*^2^ = 0.15) at *α* = 0.05.

### 2.6. Role of Funding Source

The study sponsor had no role in study design; in the collection, analysis, and interpretation of data; in the writing of the report; and in the decision to submit the paper for publication. The authors had full access to the data.

## 3. Results

### 3.1. Characteristics of Study Population

The study population comprised 121 T2DM subjects of Caucasian origin. The baseline characteristics are outlined in Table 1, whilst continuous glucose-monitoring data are shown in Table 2. Patients had reasonably well-controlled diabetes with a median HbA_1c_ of 6.8% (45 mmol/mol), a median fasting plasma glucose of 7.08 mmol/L, and a mean blood glucose (MBG) during CGM of 7.9 mmol/L. Hyperglycaemic episodes were more prevalent than hypoglycaemic episodes as shown by the number of high and low excursions, the AUC > 7.8 mmol/L and AUC< 3.9 mmol/L, as well as duration of BG > 7.8 mmol/L, between 3.9 and 7.8 mmol/L, and < 3.9 mmol/L. However, none of the study participants reported hypoglycaemic episodes, indicating that all these episodes were subclinical. There were no patients with missing data.

### 3.2. Predictors of Macrovascular Disease

**Figure 1:**
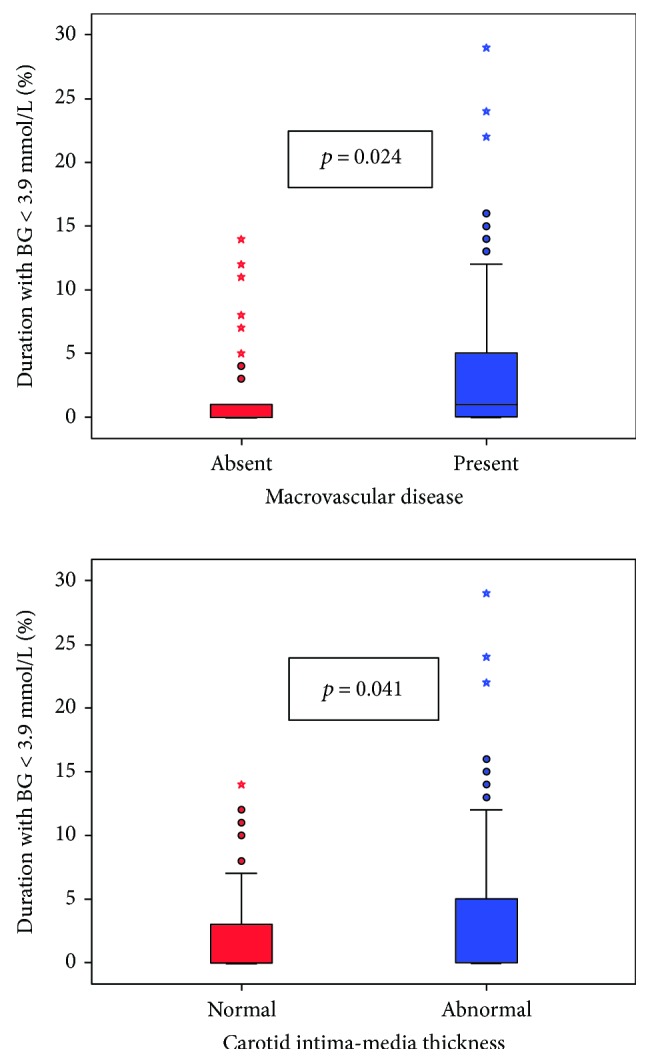
Box-and-whisker plot demonstrating relation of duration of glucose < 3.9 mmol/L (expressed as percentage of the entire 72 hr period) and macrovascular disease and carotid intima-media thickness. ^∗^denoting outliers.

Seventy-two subjects exhibited macrovascular disease. Univariate followed by multivariate analysis was performed to identify predictors of macrovascular disease in the study population. The results of univariate analysis are shown in Tables 3–5. In univariate analysis, the following variables had a *p* value < 0.1 and were thus included in the multivariate model: body surface area (BSA), smoking pack years, HDL cholesterol, triglycerides, ESR, lowest blood glucose (BG) value, AUC for BG < 3.9 mmol/L, and duration of BG < 3.9 mmol/L. In multivariate logistic regression analysis, BSA (OR 18.88 (95% CI 2.20–156.69), *p* = 0.006) and duration of BG < 3.9 mmol/L (OR 1.12 (95% CI 1.014–1.228), *p* = 0.024) were shown to be independent predictors of macrovascular disease. The relation between presence of cardiovascular disease and duration of BG < 3.9 mmol/L is shown in Figure 1.

### 3.3. Predictors of Abnormal Carotid Intima-Media Thickness

In the study population, 64 subjects had abnormal CIMT (composite of increased CIMT and presence of carotid plaque). In univariate analysis, the following variables had a *p* value < 0.1: age, DM duration, smoking pack years, BSA, white cell count, eGFR, ESR, and duration of BG < 3.9 mmol/L. These variables were consequently included in the multivariate model.

Binary logistic regression analysis revealed that BSA (OR 12.6 (95% CI 1.70–93.54), *p* = 0.013) and duration of BGM < 3.9 mmol/L (OR 1.09 (95% CI 1.003–1.187), *p* = 0.041) were independent predictors of abnormal CIMT. The relation between abnormal CIMT and duration of BG < 3.9 mmol/L is shown in Figure 1.

### 3.4. Predictors of Albuminuria

The following had *p* value < 0.1 in univariate analysis: low eGFR (<60 mL/min/1.73m^2^), white cell count, HDL cholesterol, triglycerides, uric acid, BMI, WI, MBG, SD, AUC for BG > 7.8 mmol/L, duration of BG > 7.8 mmol/L, duration of BG between 3.9 and 7.8 mmol/L, and highest BG value recorded. Generalised linear regression model revealed AUC for BG > 7.8 mmol/L (*β* = 15.83, *p* = 0.005) to be the sole independent predictor of albuminuria.

## 4. Discussion

The present study found that the duration of blood glucose < 3.9 mmol/L was significantly and independently associated with abnormal CIMT and with the composite of abnormal CIMT, ABI, coronary artery disease, and cerebrovascular disease in subjects with type 2 diabetes. This suggests that hypoglycaemia may predispose to atherosclerosis. These findings are consistent with those of the Diabetes Control and Complications Trial (DCCT), which reported that the rate of severe hypoglycaemic episodes in subjects with type 1 diabetes was associated with increased coronary calcium score in those with a HbA_1c_ below 7.5% (58 mmol/L) [18]. It is, however, possible that severe hypoglycaemia was acting as a marker for increased glucose fluctuations. Since we performed CGM, we can exclude this possibility in our study. Furthermore, whilst the DCCT data showed an association of severe hypoglycaemia with increased coronary calcium score in type 1 diabetes, the present study shows a relationship of mild asymptomatic hypoglycaemia, as detected by CGM, to atherosclerosis in type 2 diabetes.

We did not find significant associations of any of the hyperglycaemic indexes (including HbA_1c_, fructosamine, AUC > 7.8 mmol/L, and duration > 7.8 mmol/L) or of glucose fluctuations (as assessed by standard deviation of blood glucose during CGM) with macrovascular disease. However, duration of blood glucose > 7.8 mmol/L was independently associated with ACR. These observations may be related to the short median duration of diabetes in our patients. Our findings are consistent with clinical trial data which have shown that lowering blood glucose for a few years is effective in reducing microvascular but not macrovascular disease [2, 3]. In the UKPDS, it took 10 years of intensive control and a further 10 years of follow-up to demonstrate a beneficial effect of lowering blood glucose on macrovascular outcomes [20]. Another reason could be the relatively good glycaemic control in our cohort.

Our data suggest that hypoglycaemia may have a more rapid effect in promoting atherosclerosis than mild hyperglycaemia or glucose fluctuations. They may help explain the relationship between hypoglycaemia and adverse cardiovascular outcomes even in the short term and why these deleterious effects extend well beyond the period of hypoglycaemia itself. They may also explain why too aggressive lowering of blood glucose in the Action to Control Cardiovascular Risk in Diabetes (ACCORD) trial was associated with increased mortality [21] and why agents such as sulphonylureas [22, 23]and insulin [24] which are prone to cause hypoglycaemia have also been associated with increased mortality and cardiovascular events by some authors.

Hypoglycaemia can promote atherogenesis in a number of ways. Acute hypoglycaemia has been shown to result in decreased nitric oxide bioavailability, increased oxidative stress [25], platelet activation [26], and release of proinflammatory [27], proatherogenic, and prothrombotic cytokines such as PAI-1, VEGF, vascular adhesion molecules, and interleukin-6 [26, 28]. Many of these effects may extend to beyond the hypoglycaemic period [26]. Furthermore, Fadini and colleagues have recently reported that hypoglycaemia may impair endothelial progenitor cell response [29]. Interestingly, Peña and colleagues have reported that hypoglycaemia, but not glucose variability, is associated with endothelial dysfunction in children with type 1 diabetes [30].

Body surface area was also found to be independently associated with atherosclerotic markers. This is probably related to insulin resistance and/or hyperinsulinaemia.

The patients with macrovascular disease were on average younger than those without macrovascular disease. This could be due to the cross-sectional nature of our study. Macrovascular disease shortens life expectancy resulting in a lower average age of subjects with macrovascular disease compared to subjects without. Furthermore, macrovascular disease was associated with hypoglycaemia, which is also known to decrease survival [21]. Duration of diabetes was similar in both groups, meaning that those with macrovascular disease had younger onset type 2 diabetes, which might be associated with higher mortality [31].

A limitation of our study is that we have no information on antecedent glycaemic indexes. This problem is inherent to the study area as it is not practical or ethical to perform CGM over a number of years and not alter management in order to keep glycaemic parameters stable. However, we tried to reduce the impact of this limitation in a number of ways. Firstly, we only recruited patients with stable control, namely, not needing escalation of treatment. Secondly, we studied patients with short duration of diabetes because such patients are less likely to have variation in their glycaemic parameters over the course of their disease. Thirdly, we performed blind, rather than real-time, CGM so that patients did not alter their behaviour as result of the glucose readings. Additionally, patients were instructed not to change their usual routines. In this way, the CGM data are more likely to be representative of the patients' usual glycaemic profile. Furthermore, many glycaemic indices such as HbA_1c_ [32] and hypoglycaemia [11] have been shown to exhibit significant temporal tracking. Another limitation is that in view of the cross-sectional nature of our study, we cannot prove causal relationships. It is possible that those with macrovascular disease were treated more aggressively, hence causing more hypoglycaemia. However, this would not explain the observed relationship with CIMT, which is a subclinical marker of atherosclerosis.

In conclusion, the study suggests that mild asymptomatic hypoglycaemia in subjects with type 2 diabetes is associated with atherosclerotic disease. This cannot be explained by an association of hypoglycaemia with hyperglycaemia or glucose fluctuations. Hyperglycaemic load, as measured by AUC for BG > 7.8 mmol/L, was associated with microalbuminuria.

## Figures and Tables

**Table 1:** Baseline demographic and clinical findings of study population.

Patient characteristics (*n* = 121)	Values
Age (years)^†^	64 (57–68)
Male : female (*n* (%))	89 (73.6) : 32 (26.4)
Diabetes duration (years)^†^	3 (2–5)
Smoking (current/ex/nonsmokers) (*n* (%))	22 (18.2)/41 (33.9)/58 (47.9)
Hypertension (*n* (%))	80 (66.1)
Hyperlipidaemia (*n* (%))	87 (71.9)
Ischaemic heart disease (*n* (%))	15 (12.4)
Peripheral arterial disease (*n* (%))	7 (5.8)
Cerebrovascular accident/transient ischaemic attack (*n* (%))	3 (2.5)
Abnormal carotid intima-media thickness (*n* (%))	64 (52.9)
Macrovascular disease (*n* (%))	72 (59.5)
Chronic kidney disease (*n* (%))	9 (7.4)
On metformin (*n* (%))	109 (90.1)
On sulphonylurea (*n* (%))	36 (29.8)
On gliptins (*n* (%))	6 (5)
On insulin (*n* (%))	6 (5)
On angiotensin-converting enzyme inhibitor/on angiotensin receptor blocker (*n* (%))	77 (63.6)
On *β*-blocker (*n* (%))	22 (18.2)
On calcium channel blocker (*n* (%))	23 (19)
On diuretic (*n* (%))	33 (23.7)
On aspirin (*n* (%))	38 (31.4)
On statin (*n* (%))	81 (66.9)
On fibrate (*n* (%))	7 (5.8)
On allopurinol (*n* (%))	5 (4.1)
Body mass index (kg/m^2^)^†^	31.22 (27.94–34.53)
Body surface area (m^2^)^∗^	1.89 ± 0.21
Waist index^∗^	1.19 ± 0.14
Pulse rate (bpm)^†^	68 (61.5–78.5)
Mean systolic pressure (mmHg)^∗^	144.19 ± 17.82
Mean diastolic pressure (mmHg)^∗^	84.60 ± 8.76
Pulse pressure (mmHg)^∗^	61.53 ± 13.66
Mean arterial pressure (mmHg)^†^	107 (93–113)
White cell count (×10^9^/L)^†^	7.16 (6.26–8.69)
Haemoglobin (g/dL)^†^	14.35 (13.33–15.18)
Platelet count (×10^9^/L)^†^	240.5 (206–284.75)
Red blood cell distribution width (%)^†^	13.1 (12.53–13.7)
Mean platelet volume (fL)^†^	10.9 (10.2–11.6)
Estimated glomerular filtration rate (mL/min/1.73 m^2^)^∗^	91.22 ± 24.61
Total cholesterol (mmol/L)^†^	4.12 (3.55–5.15)
HDL cholesterol (mmol/L)^†^	1.24 (1.06–1.51)
LDL cholesterol (mmol/L)^†^	2.19 (1.67–3.19)
Non-HDL cholesterol (mmol/L)^†^	2.8 (2.26–3.82)
Triglycerides (mmol/L)^†^	1.36 (0.97–1.7)
Alkaline phosphatase (mmol/L)^†^	69 (55–83.5)
Alanine transaminase (mmol/L)^†^	23 (18–32.5)
Uric acid (*μ*mol/L)^∗^	313.05 ± 79.98
Erythrocyte sedimentation rate (mm 1st hr)^†^	10.5 (6–18)
Albumin-creatinine ratio (mg/mmol)^†^	7.08 (1–23.34)
Fasting plasma glucose (mmol/L)^†^	7.08 (6.11–8.18)
Fructosamine (*μ*mol/L)^†^	278 (257–303)
Glycated haemoglobin (%)^†^	6.8 (6.3–7.6)

Values are expressed as number (%) of patients, ^∗^mean ± SD, or ^†^median (IQR).

**Table 2:** Baseline continuous glucose-monitoring findings of study population.

Patient characteristics (*n* = 121)	Values
Highest value (mmol/L)	13.2 (11.3–15.5)
Lowest value (mmol/L)	3.95 (2.93–5.1)
Mean blood glucose (mmol/L)	7.35 (6.63–8.65)
Standard deviation (mmol/L)	1.9 (1.5–2.3)
Number of high excursions	8 (5–10)
Number of low excursions	0 (0–2)
AUC above 7.8 mmol/L	0.63 (0.23–1.43)
AUC below 3.9 mmol/L	0 (0–0.02)
Duration during 72 hr period with BG above 7.8 mmol/L (%)	37 (19.25–60.75)
Duration during 72 hr period with BG within 3.9–7.8 mmol/L (%)	60 (37.5–77)
Duration during 72 hr period with BG below 3.9 mmol/L (%)	0 (0–4)

Values are expressed as median (IQR). AUC: area under curve.

**Table 3:** Demographic and clinical findings of subjects with and without macrovascular disease.

Variable	No macrovascular disease (*n* = 49)	Macrovascular disease (*n* = 72)	*p* value
Age (years)^†^	66 (62–69)	61 (54.25–67)	0.001
Male : female (*n* (%))	34 **(69.4)** : 15 **(**30.6)	55 **(76.4)** : 17 **(23.6)**	0.41
Diabetes duration (years)^†^	3.5 (2–5)	3 (2–4)	0.23
Smoking (current/ex/nonsmokers) (*n* (%))	4 (8.1)/19 (38.8)/26 (53.1)	18 (25.0)/22 (30.6)/32 (44.4)	0.06
Hypertension (*n* (%))	29 (59.18)	51 (70.83)	0.24
Hyperlipidaemia (*n* (%))	36 (73.47)	51 (70.83)	0.84
CKD (*n* (%))	2 (4.08)	7 (9.72)	0.31
Metformin (*n* (%))	44 (89.80)	65 (90.28)	1.00
Sulphonylurea (*n* (%))	14 (28.57)	50 (69.44)	0.84
Gliptins (*n* (%))	4 (8.16)	2 (2.77)	0.22
Insulin (*n* (%))	1 (2.04)	5 (6.94)	0.40
ACEI/ARB (*n* (%))	29 (59.18)	48 (66.67)	0.44
*β*-blocker (*n* (%))	7 (14.29)	15 (20.83)	0.47
CCB (*n* (%))	11 (22.45)	12 (16.67)	0.48
Diuretic (*n* (%))	14 (28.57)	19 (26.39)	0.84
Aspirin (*n* (%))	9 (18.36)	29 (40.28)	0.02
Statin (*n* (%))	29 (59.18)	52 (72.22)	0.17
Fibrate (*n* (%))	6 (12.24)	1 (1.39)	0.02
Allopurinol (*n* (%))	2 (4.08)	3 (4.17)	1.00
BMI (kg/m^2^)^†^	31.22 (27.67–33.97)	31.31 (27.9–35.19)	0.35
BSA (m^2^)^∗^	1.83 ± 0.20	1.93 ± 0.21	0.007
Waist index^∗^	1.17 ± 0.15	1.20 ± 0.13	0.22
Pulse rate (bpm)^†^	65 (61–80.25)	71 (62–78)	0.84
Mean SBP (mmHg)^∗^	146 ± 18.28	142.96 ± 17.54	0.36
Mean DBP (mmHg)^∗^	85.66 ± 7.34	83.88 ± 9.58	0.25
Pulse pressure (mmHg)^∗^	62.18 ± 15.6	61.07 ± 12.22	0.68
MAP (mmHg)^†^	106 (97.5–112.5)	90.75 (107.5–113)	0.77

Values are expressed as number (%) of patients, ^∗^mean ± SD, or ^†^median (IQR). ACEI: angiotensin-converting enzyme inhibitor; ARB: angiotensin receptor blocker; BMI: body mass index; BSA: body surface area; CCB: calcium channel blocker; CKD: chronic kidney disease; CVD: cardiovascular disease; DM: diabetes mellitus; DBP: diastolic blood pressure; MAP: mean arterial pressure; SBP: systolic blood pressure.

**Table 4:** Laboratory findings of subjects with and without macrovascular disease.

Variable	No macrovascular disease (*n* = 49)	Macrovascular disease (*n* = 72)	*p* value
White cell count (×10^9^/L)^†^	7.09 (5.88–7.87)	7.29 (6.42–9.24)	0.13
Haemoglobin (g/dL)^†^	14.2 (13.3–15.1)	14.4 (13.6–15.2)	0.48
Platelet count (×10^9^/L)^†^	242 (204.5–283.5)	239 (206–287)	0.78
RDW (%)^†^	13 (12.5–13.45)	13.1 (12.6–13.8)	0.13
MPV (fL)^†^	10.95 (10.23–11.5)	10.9 (10.1–11.6)	0.53
eGFR (mL/min/1.72 m^2^)^∗^	87.76 ± 22.21	93.58 ± 26.0	0.19
Total cholesterol (mmol/L)^†^	4.14 (3.65–5.25)	4.1 (3.53–5.11)	0.80
HDL cholesterol (mmol/L)^†^	1.32 (1.1–1.58)	1.24 (1.04–1.46)	**0.07**
LDL cholesterol (mmol/L)^†^	2.3 (1.65–3.36)	2.18 (1.7–3.13)	0.81
Non-HDL cholesterol (mmol/L)^†^	2.74 (2.18–3.86)	2.83 (2.29–3.78)	0.67
Triglycerides (mmol/L)^†^	1.13 (0.91–1.64)	1.47 (1.14–1.85)	**0.03**
ALP (mmol/L)^†^	66 (52–93)	69 (59.25–80)	0.89
ALT (mmol/L)^†^	22 (17.5–32)	24 (18–32.75)	0.6
Uric acid (*μ*mol/L)^∗^	319.56 ± 83.19	308.65 ± 78.03	0.47
ESR (mm 1st hr)^†^	13 (7–23)	9 (5–17)	**0.08**
FPG (mmol/L)^†^	7.14 (6.02–8.53)	7.08 (6.23–8.05)	0.55
Fructosamine (*μ*mol/L)^†^	281 (259.25–305)	277 (256–303.5)	0.77
HbA_1c_ (%)^†^	7.0 (6.3–8.1)	6.7 (6.3–7.4)	0.23
HbA_1c_ (mmol/L)^†^	53 (45–65)	50 (45–57)	0.23

Values are expressed as ^∗^mean ± SD or ^†^median (IQR). Abbreviations: ALP: alkaline phosphatase; ALT: alanine transaminase; eGFR: estimated glomerular filtration rate; ESR: erythrocyte sedimentation rate; FPG: fasting plasma glucose; HbA_1c_: glycated haemoglobin; MPV: mean platelet volume; RDW: red blood cell distribution width.

**Table 5:** Continuous glucose-monitoring findings of subjects with and without macrovascular disease.

Variable	No macrovascular disease (*n* = 49)	Macrovascular disease (*n* = 72)	*p* value
Highest value (mmol/L)^†^	13.4 (11.3–15.85)	13.0 (11.3–15)	0.35
Lowest value (mmol/L)^†^	4.4 (3.2–5.25)	3.7 (2.8–5)	**0.09**
Mean blood glucose (mmol/L)^†^	7.6 (6.85–9.0)	7.3 (6.6–8.4)	0.3
Standard deviation (mmol/L)^†^	3.0 (1.3–2.3)	1.8 (1.5–2.4)	0.9
Number of high excursions^†^	8 (6–10)	8 (5–10)	0.49
Number of low excursions^†^	0 (0–2)	1 (0–2)	0.10
AUC above 7.8 mmol/L^†^	0.64 (0.24–1.76)	0.57 (0.21–1.35)	0.39
AUC below 3.9 mmol/L^†^	0 (0–0.01)	0 (0–0.03)	**0.05**
Duration during 72 hr period with BG above 7.8 mmol/L (%)^†^	37 (18.5–66.5)	35 (20–58)	0.39
Duration during 72 hr period with BG within 3.9–7.8 mmol/L (%)^†^	58 (31.5–79)	60 (41–77)	0.63
Duration during 72 hr period with BG below 3.9 mmol/L (%)^†^	0 (0-1)	1 (0–5)	**0.02**

Values are expressed as number (%) of patients or ^†^median (IQR). AUC: area under curve.

## Data Availability

The data used to support the findings of this study are available from the corresponding author upon request.
